# Influence of indications on perinatal outcomes after radio frequency ablation in complicated monochorionic pregnancies: a retrospective cohort study

**DOI:** 10.1186/s12884-020-03530-6

**Published:** 2021-01-09

**Authors:** Hongmei Wang, Qian Zhou, Xietong Wang, Jia Song, Pengzheng Chen, Yanyun Wang, Lei Li, Hongyan Li

**Affiliations:** 1 Department of Obstetrics, Shandong Provincial Hospital Affiliated to Shandong First Medical University, Jinan, Shandong China; 2 Department of Obstetrics, Maternal Child Health Care Hospital of Shandong Province, Jinan, Shandong China; 3 Key Laboratory of Birth Regulation and Control Technology of National Health Commission of China, Maternal Child Health Care Hospital of Shandong Province, Jinan, Shandong China; 4 Department of Neonatology, Shandong Provincial Hospital Affiliated to Shandong First Medical University, Jinan, Shandong China

**Keywords:** Complicated monochorionic pregnancy, Intrauterine foetal death, Radiofrequency ablation, Selective foetal reduction, Selective intrauterine growth restriction, Twin–twin transfusion syndrome, Twin reversed arterial perfusion sequence, Umbilical cord occlusion

## Abstract

**Background:**

Radiofrequency ablation (RFA) is recommended to prevent potential neurological injury or intrauterine foetal death (IUFD) of the co-twin(s) in complicated monochorionic (MC) pregnancies. However, the impacts of various indications on the pregnancy outcome following RFA remain unclear. This study aimed to determine how the indications influence the perinatal outcomes in complicated MC pregnancies undergoing radiofrequency ablation.

**Methods:**

This was a retrospective cohort study performed in a single centre. All consecutive MC pregnancies treated with RFA between July 2011 and July 2019 were included. The adverse perinatal outcomes and the survival rate were analysed based on various indications. The continuous variables with and without normal distribution were compared between the groups using Student’s t-test and Mann–Whitney U test, respectively, and for categorical variables, Chi-square and Fisher’s exact tests were used. P < 0.05 indicated a significant difference.

**Results:**

We performed 272 RFA procedures in 268 complicated MC pregnancies, including 60 selective intrauterine growth restriction (sIUGR), 64 twin–twin transfusion syndrome (TTTS), 12 twin reversed arterial perfusion sequence (TRAPs), 66 foetal anomaly and 66 elective foetal reduction (EFR) cases. The overall survival rate of the co-twin was 201/272 (73.9%). The overall technical successful rate was determined at 201/263 (76.7%). The IUFD rate in the co-twin was 20/272 (7.4%). The TTTS group had recorded the lowest survival rate (37/64, 57. 8%), and the survival rate was significantly correlated with Quintero stages (P = 0.029). Moreover, the sIUGR III subgroup had a lower survival rate compared with sIUGR II (55.6%, versus 84.3%). The subgroup of foetal anomaly of gastroschisis or exomphalos had the highest IUFD rate (4/10, 40%), followed by sIUGR III (2/9, 22.2%) and dichorionic triamniotic (DCTA) subgroup (8/46, 17.9%). In EFR group, eight IUFD cases were all coming from the DCTA subgroup and received RFA before 17 weeks.

**Conclusions:**

The perinatal outcome of RFA was correlated with the indications, with the lowest survival rate in TTTS IV and the highest IUFD incidence in abdominal wall defect followed by sIUGR III. Elective RFA after 17 weeks may prevent IUFD in DCTA pregnancies.

## Background

Around one-third of the monochorionic (MC) multiple pregnancies suffer from specific complications such as twin–twin transfusion syndrome (TTTS), selective intrauterine growth restriction (sIUGR), twin reversed arterial perfusion sequence (TRAPs) and twin anaemia polycythaemia sequence (TAPS) [1, 2]. For complicated MC pregnancies, selective foetal reduction by umbilical cord occlusion (UCO) is recommended to prevent potential neurological injury or intrauterine foetal death (IUFD) of the remaining foetus [3–5].

UCO techniques include laser cord coagulation [6], cord ligation [7], bipolar cord coagulation [4, 7], radiofrequency ablation (RFA) [8–10], microwave ablation [11] and high-intensity focused ultrasound [12]. The optimal method of UCO remains to be determined. RFA, using a 17-gauge needle to ablate tissues within 2 cm diameter, is relatively simple and effective, obtaining a lower incidence of preterm premature rupture of membranes (PPROM) and preterm delivery [5, 8, 13]. Other advantages that make RFA the preferred technique involve situations where there is difficulty in terms of access to other surgical treatments, including oligohydramnios of the target twin, proximity of twin cord insertion sites, anterior placenta or earlier gestational age [14].

The primary indications for RFA are TRAPs and other lethal foetal anomalies [14]. After RFA, the overall survival rate of the co-twin in TRAPs is determined to be at 75.0–92% [8–10, 15–19], but it varies in other anomalies, ranging 33–92.3% [8, 13, 15]. RFA is also recommended for sIUGR in which laser may not be a desirable choice for large vessel anastomosis [5, 20, 21]. Although foetoscopic laser photocoagulation (FLP) is currently the standard treatment for TTTS stages II–IV [22–24], selective foetal reduction is still required, because this technique is still not available in many areas, or the surgeons are not experienced, especially in patients with an anterior placenta. At times, parents prefer to undergo RFA owing to concerns regarding the foetal outcome. Maternal reasons included history of caesarean section or cervical incompetence, and increased maternal age [25, 26].

However, the effects of these indications on the pregnancy outcome following RFA remain undefined. Therefore, by examining a large cohort of complicated MC pregnancies undergoing RFA, we determined how the various indications influenced the perinatal outcome after RFA treatment.

## Methods

### Study population

This retrospective cohort study enrolled all cases of consecutive MC pregnancies treated with RFA in a single tertiary centre between July 2011 and July 2019. The indications for RFA were as follows: sIUGR II and III [27]; TTTS stages III–IV [28]; MC twins discordant for foetal anomaly (MTDFA); TRAPs (abdominal circumference of the acardiac twin larger than that of the pump twin) and elective foetal reduction (EFR) for multiple pregnancies containing MC components. We also included those patients with TTTS II who chose foetal reduction of an MC co-twin for maternal reasons. Conversely, RFA contraindications were as follows: < 2.5 cm cervical length, uterine contraction, vaginal bleeding or severe maternal complications. The ethics committee of Shandong Provincial Hospital affiliated to Shandong First Medical University has approved this study, and the registry number is 2011-023.

### Description of procedure

Patients underwent a comprehensive ultrasound examination to determine the chorionicity status, placental position, malformations, amniotic fluid’s deepest vertical pocket, cervical length, umbilical artery blood flow and the middle cerebral artery peak systolic velocity (MCA–PSV). Patients and their families received detailed counselling regarding the risks of RFA, including co-twin demise, miscarriage, preterm labour and neurological or thermal injury to the surviving foetus. Written consent forms were signed by patients.

All RFA procedures were performed under local anaesthesia, and either supine or lateral position was used depending on the convenience of access to the targeted vessel. Under ultrasound guidance, a 17-gauge Sarburst radiofrequency needle (RITA Medical Systems, Fremont, CA, USA) was inserted to get near to the abdominal segment of the foetal umbilical vessel, trying to avoid the placenta and amniotic sac of the retained foetus(es). The accuracy of the ablation area is essential for the success of the RFA procedure. If the umbilical vessels could not be surrounded by the tines, the position of the needle was adjusted.

Three electrodes were ejected to surround the cord vessels after the tine position was confirmed. Radiofrequency energy was applied for 3 min at 150 watts to generate the target temperature of 100 °C–110 °C. If the three electrode tines did not exhibit a synchronous temperature increase (difference: >30 °C), they were retracted, and the position was then adjusted. During and after each ablation cycle, the blood flow in the targeted vessels was observed using the colour Doppler until the blood flow signal was completely absent. The heart rate of the targeted foetus was then monitored until it decreased to < 60 bpm. However, the disappearance of the blood flow signal in the umbilical vessels does not always indicate a complete blockage; it may be merely too weak to be detected after a reflective decrease of the heart rate and output or spasm of the vessel. If the heartbeat stopped earlier or the decrease of the heartbeat was slower than expected, another ablation cycle was planned.

An ultrasound examination was performed 24 h after the procedure to evaluate the surviving foetus, the systolic/diastolic pressure of the umbilical artery and MCA–PSV and cervical length. Thereafter, further ultrasound scan was performed every 2 weeks. MRI was also performed at 24–28 weeks to detect any foetal brain damage. Subsequently, the patients were allowed to consult and deliver in local hospitals.

### Data collection

The following data of all the consecutive patients who underwent RFA were retrieved from our database: maternal age, indications for foetal reduction, gestational age at procedure, postprocedural complications, subsequent ultrasound scan and foetal brain MRI results. Furthermore, the following perinatal outcomes were also recorded: IUFD of the co-twin, PPROM within 2 weeks after the RFA, termination of pregnancy (TOP) and preterm delivery < 28 weeks, gestational age at delivery and birth weight and perinatal mortality. In cases delivered elsewhere, we gathered the delivery and neonatal information, and a neonatologist evaluated the condition of the newborns according to the Gessel Developmental Schedule [29], both via a telephone interview with the parents.

### Statistical analysis

Patients were divided into five groups based on their conditions, namely, sIUGR, TTTS, TRAPs, MTFDA and EFR. They were further split into subgroups according to the type of sIUGR, Quintero stage of TTTS, position of foetal anomaly and the chorionicity of EFR. The incidence rate was calculated per procedure because some patients had undergone two procedures. The survival rate was calculated when the co-twin(s) were delivered after 28 weeks and the newborn was alive at 28 days. Technical successful rate was calculated to exclude those TOP cases after RFA because of foetal anomaly or other reasons and those who gave up further treatment on the premature newborns. Take-baby-home rate per patient was also calculated because most triplet pregnancies receiving RFA were successful despite the co-twin demise.

Data were analysed using the SPSS version 25.0 software (IBM SPSS Statistics). Continuous variables are presented as mean and standard deviation, whereas categorical variables are expressed as absolute numbers and percentages. The continuous variables with and without normal distribution were compared between the groups by Student’s *t*-test and Mann–Whitney *U* test, respectively; for categorical variables, we used the chi-square and Fisher’s exact tests. Differences were considered significant when *P* < 0.05.

## Results

Table 1 presents the characteristics and the overall pregnancy outcomes for the total population and the five groups. During the study period, 272 RFA procedures were performed in 268 patients; two procedures were performed in one patient including 2 TRAPs [1 monochorionic tramniotic (MCTA) and 1 monochorionic quadramniotic (MCQA)], 1 MCQA with foetal anomaly, 1 DCQA with EFR. We found 60 sIUGR, 64 TTTS, 12 TRAPs, 66 foetal anomaly and 70 EFR cases. The median gestational age at procedure in the total population was determined to be at 20.05 ± 3.41 weeks, and that in the EFR group (16.73 ± 1.31 weeks) was significantly the earliest among the five groups (*P* < 0.001). The EFR group achieved the best pregnancy outcome followed by the sIUGR group, with the same co-twin survival rate (80.0%) and a higher take-baby-home rate in the EFR group (85.1%). The overall survival rate of the co-twin was determined to be 201/272 (73.9%). The median gestational age at delivery was 36.3 ± 2.9 weeks, with an average birth weight of 2662.5 ± 707.8 g. After the exclusion of those who opted for TOP (*n* = 7) and who gave up on postnatal treatment (*n* = 2), the overall technical successful rate was 201/263 (76.4%).

**Table 1:** Characteristics and perinatal outcomes in complicated multiple pregnancies undergoing RFA

Characteristic	sIUGR (*n* = 60)	TTTS (*n* = 64)	TRAPs (*n* = 12)	Foetal anomaly(*n* = 66)	EFR(*n* = 70)	Sum (*n* = 272)	P
Age pregnancy	31.17±4.82	30.83±4.64	30.33±5.68	30.85±4.86	31.10±4.57	30.96±4.73y	0.982
GA at procedure	22.04±2.77	21.17±2.77	21.42±4.22	20.45±3.51	16.73±1.31	20.05±3.41w	**<0.001**
IUFD	2(3.3%)	3(4.7%)	0	7(10.6%)	8(11.4%)	20(7.4%)	0.217
PROM within 2w	5(8.3%)	5(7.8%)	1(8.3%)	2(3.0%)	1(1.4%)	14(5.1%)	0.286
TOP	0	5(7.8%)	1(8.3%)	1(1.5%)	0	7(2.6%)	**0.015**
Miscarriage (> 2w after RFA)	3(5.0%)	11(17.2%)	1(8.3%)	5(7.6%)	5(7.1%)	25(9.2%)	0.151
Delivery at 28–31^+ 6^w	4(6.7%)	4(6.3%)	0	2(3.0%)	0	10(3.7%)	0.205
Delivery at 32–33^+ 6^w	7(11.7%)	12(18.8%)	4(33.3%)	12(18.2%)	25(35.7%)	60(22.1%)	**0.010**
Delivery at 37w-	37(61.7%)	21(32.8%)	5(41.7%)	37(56.1%)	31(44.3%)	131(48.2%)	**0.013**
GA at delivery(w)	36.58±3.28	35.35±3.72	36.46±2.60	36.93±2.61	36.29±1.92	36.34±2.90w	0.265
Birth weight	2983.80±797.30	2459.25±777.58	2731.11±599.45	2768.24±669.14	2413.39±463.36	2662.48±707.80	**<0.001**
Technical success	48(80.0%)	37(64.9%)	9(81.8%)	51(78.5%)	56(80.0%)	201(76.4%)	0.245
Neonatal death	2(3.3%)	3(4.7%)#	0	0	0	5(1.8%)#	0.341
Survival rate of co-twin	48(80.0%)	37(57.8%)	9(75.0%)	51(77.3%)	56(80.0%)	201(73.9%)	**0.022**
Take-baby-home rate	48(80.0%)	37(57.8%)	9(75.0%)	51(77.3%)	57/67(85.1%)	202/268(75.3%)	**0.020**

Data are presented as mean ± SD, n (%).#Treatment was given up by the parents for worrying about the potential longterm developmental retardation

IUFD of the co-twin occurred in 20/272 (7.4%) procedures of the total population and 17/20 (85.0%) procedures within 2 days after RFA. The incidence of premature rupture of membranes (PROM) within 2 weeks after RFA was at 14/272 (5.1%). Elective TOP was preferred by parents for dysplasia in the co-twin including severe oedema (*n* = 3) and tricuspid insufficiency (*n* = 2) in the TTTS group and cleft palate in the MTDFA group (*n* = 1) and of maternal complication (*n* = 1) in the TRAPs group. The highest IUFD rate was found in the EFR group (8/70, 11.4%), followed by the MTDFA group (7/66, 10.6%), whereas the lowest IUFD rate was found in the TRAPs group (0/12, 0%), followed by the sIUGR group (2/60, 3.33%). Meanwhile, the lowest survival rate of 37/64 (57.8%) was recorded in the TTTS group, considering the three IUFD (4.7%) and five PROM (7.8%) cases within 2 weeks after the procedure, five TOP cases for tricuspid insufficiency and severe hydrops foetalis and the highest miscarriage rate.

The pregnancy outcomes stratified by the types of sIUGR, Quintero stages of TTTS, position of foetal anomaly in the MTDFA group and chorionicity of EFR are presented in Table 2. The survival rate of the subgroup of TTTS stages II, III and IV was 100%, 63.9% and 36.4%, respectively, and is significantly correlated with Quintero stages (*P* = 0.029). The technical successful rate in sIUGR III (5/9, 55.6%) was lower than that in sIUGR II (43/51, 84.3%), but the difference was not significant, probably owing to the small sample size of the sIUGR III subgroup (*P* = 0.069). The highest IUFD rate (4/10, 40%) was found in the subgroup of foetal anomaly in the abdominal wall (gastroschisis or exomphalos), followed by the subgroups of sIUGR III (2/9, 22.2%) and dichorionic triamniotic (DCTA) for EFR (8/46, 17.4%).

**Table 2:** Perinatal outcomes after RFA in complicated monochorionic pregnancies stratified by indications

	Subgroups	IUFD	2W PROM	Miscarriage (> 2w after RFA*)	TOP	Neonatal death	Technical success
sIUGR (*n* = 60)	II (*n* = 51)	0	4 (7.8%)	3 (5.9%)	0	1 (2.0%)	43/51 (84.3%)
	III (*n* = 9)	2 (22.2%)	1 (11.1%)	0	0	1 (11.1%)	5/9 (55.6%)
	P	0.020	0.570	1.000	-	0.280	0.069
TTTS (*n* = 64)	II (*n* = 11)	0	0	0	0	0	10 (100.0%)
	III (*n* = 39)	2 (5.1%)	5 (12.8%)	5 (12.8%)	2 (5.1%)	1 (2.6%)	23 (63.9%)
	IV (*n* = 14)	1 (7.1%)	0	6 (42.9%)	3 (21.4%)	0	4 (36.4%)
	P	0.688	0.176	0.010	0.085	0.722	0.009
TRAPs (*n* = 12)	TRAP (*n* = 12)	0	1 (8.3%)	1(8.3%)	1 (8.3%)	0	9 (81.8%)
Discordant for foetal anomaly (*n* = 66)	Central neural system (*n* = 20)	0	1 (5.0%)	2(10.0%)	1 (5.0%)	0	16 (84.2%)
	Heart (*n* = 10)	2 (20%)	0	0	0	0	8 (80.0%)
	Edema (*n* = 13)	0	1 (7.7%)	2 (15.4%)	0	0	10 (76.9%)
	Gastrochisis, Exompholos (*n* = 10)	4 (40%)	0	0	0	0	6 (60.0%)
	Other (*n* = 13)	1 (7.7%)	0	1 (7.7%)	0	0	11 (84.6%)
	P	0.007	0.689	0.568	0.674	-	0.607
EFR (*n* = 70)	DCTA (*n* = 46)	8 (17.4%)	0	3 (6.5%)	0	0	35 (76.1%)
	MCTA (*n* = 17)	0	1 (5.9%)	2 (11.8%)	0	0	14 (82.4%)
	Other (*n* = 7)	0	0	0	0	0	7 (100.0%)
	P	0.095	0.181	0.513	-	-	0.293
Sum (*n* = 272)	P	<0.001	0.470	0.015	0.009	0.586	0.020

Data are presented as mean ± SD, n (%)

**Fig. 1:**
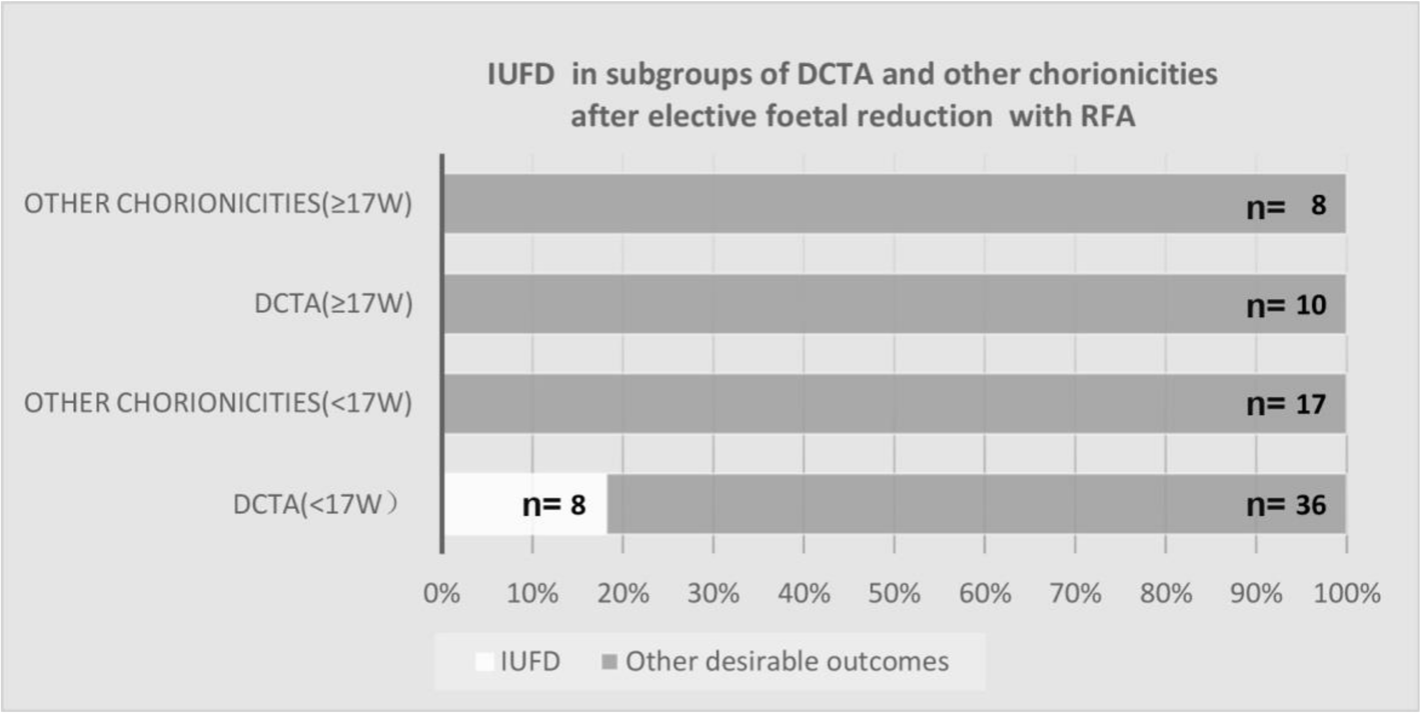
IUFD after elective foetal reduction with RFA in subgroup of DCTA and other chorionities

The EFR group involved 46 DCTA, 17 MCTA, 1 DCQA (2 procedures in one patient), and 5 MCDA twin pregnancies choosing foetal reduction. With no IUFD after RFA, the pregnancy outcome in the MCTA subgroup was found to be slightly better than that in the DCTA subgroup (81.3% vs. 76.1%, *P* = 0.607). The eight IUFD cases were all from the DCTA subgroup and received RFA before 17 weeks; for those who received RFA after 17 weeks (*n* = 19), they all ended with desirable outcomes regardless of the chorionicity (Fig. 1).

During the RFA procedure, 25 patients presented with supine hypotension, which was relieved after changing the body position and providing oxygen inhalation. No severe maternal complication such as deep vein thrombosis or burn in the skin was found. Only one patient exhibited iatrogenic septostomy 5 days after RFA, with no adverse outcome. Furthermore, one patient experienced chorioamnionitis 9 days after RFA, followed by TOP.

Table 3 presents other postprocedural pregnancy complications. TTTS was found to occur in two patients with MCTA; one underwent abortion at 25^+ 6^ weeks after serial amnioreduction, whereas the other one delivered at 32^+ 4^ weeks. Placenta abruption, preeclampsia and acute pancreatitis were also found to occur in late pregnancy, but with timely caesarean section, the outcome was successful.

**Table 3:** Other perinatal complications may affect the pregnancy outcome after RFA

Complications	Chorionicity	Indication for RFA	GA at delivery (week)	Prolonged GA (week)	Pregnancy outcome
Pre-eclampsia	DCTA	EFR	36^+ 4^	20^+ 2^	3070 g/2990 g
Acute pancreatitis	DCTA	EFR	29^+ 4^	13^+ 5^	1400 g/1300 g
Placental abruption	MCDA	sIUGRII	34^+ 3^	10^+ 3^	2300 g
Placental abruption	MCDA	sIUGRIII	32^+ 4^	6^+ 6^	3000 g
TTTS	MCTA	EFR	25^+ 6^	6^+ 3^	Abortion after amnioreduction
TTTS	MCTA	EFR	32^+ 4^	13^+ 2^	1760 g/2020 g

None of the surviving co-twins showed increased MCA–PSV at follow-up with ultrasound, or abnormal foetal brain MR, except for mild ventriculomegaly in one pregnancy (1.2 cm). Treatment of the two preterm newborns delivered at 27^+ 2^ weeks and 28^+ 2^ weeks was stopped by their parents as they were worried about the potential neurodevelopmental retardation. One delivered at 30^+ 5^ weeks with severe infection, and two preterm newborns delivered at 28^+^ weeks died despite receiving active treatment in local hospitals. Nonetheless, 6/10 neonates delivered between 28 and 32 weeks were doing well after NICU admission. In the follow-up interview, all children met the main criteria based on their age, and none of the parents complained about any developmental problems of their children.

## Discussion

This study investigated the perinatal outcome of 268 MC pregnancies undergoing 272 RFA procedures, and the overall survival rate was 73.9% in the co-twins. Postprocedural complications of RFA include thermal injuries [5, 8], IUFD and brain damage to the retained co-twin [8, 21, 30] and preterm birth [16, 31]. However, the effects of various indications on the adverse perinatal outcomes after RFA are still poorly understood. In this study, considering the large cohort size, we could analyze the pregnancy outcomes stratified by the indications of TTTS, sIUGR, TRAPs, discordant anomaly and EFR, and further investigate how the types of sIUGR, Quintero stages of TTTS, the position of foetal anomaly and the chorionicity of EFR contributed to the perinatal outcome after RFA.

The perinatal outcome was found to be significantly different with respect to various indications. Regarding the survival rate, the lowest was recorded in the TTTS group, whereas the highest was in the EFR and sIUGR groups, conforming to previous studies [8, 13, 15, 19, 25]. Kumar [8] and Yinon [19] reported a survival rate of 38/38 and 17/19 in the sIUGR group separately and that sIUGR as an indication for RFA had a more favourable perinatal outcome than other indications. In the current study, the poor perinatal outcome in the TTTS group and the unexpectedly lower survival rate in the MTDFA group could be explained by further subgroup analysis.

The perinatal outcomes were found to be significantly correlated with the Quintero stages of TTTS, which was worst in TTTS IV, having higher incidences of polyhydramnios and miscarriage, and selective termination for severe tricuspid insufficiency or oedema in the retained foetus. The sIUGR II subgroup was able to obtain a better survival rate than the sIUGR III subgroup, probably contributed by the higher incidence of IUFD in the subgroup sIUGR III, which involves larger artery–artery anastomosis [1, 25], allowing more blood exchange during ablation.

Additionally, two subgroups had high IUFD rates. As for the high rate of 40% in the subgroup of foetal anomaly in the anterior abdominal wall (gastroschisis or exomphalos), with the proximity of the target vessel and the heart of the targeted twin, we speculate that the disappearance of the cord blood flow during RFA might be the consequence of the damage to the heart caused by ablation energy rather than a complete blockage of the blood flow, and subsequent exsanguination resulted in the co-twin demise. This finding provided an evidence to consider other UCO techniques in similar situations. Furthermore, an unexpected higher rate of 8/46 was found in the DCTA subgroup, with all IUFD cases occurred in those performed before 17 weeks, wherein technical difficulty was worsened by the small size of the targeted foetus. Meanwhile, IUFD did not occur in the MCTA subgroup with the same gestational age, probably because of the flexible option in any of the three foetuses, making the process technically simpler than in DCTA cases wherein only two of the three foetuses could be targeted. Technical difficulty and subsequent IUFD in the DCTA subgroup could be partially avoided by performing the procedure after 17 weeks.

The accurate mechanism of the co-twin demise remains unclear. Subsequent exsanguination caused by an incomplete blockage in the targeted vessel after ablation might be a reason [4]. In this study, no IUFD in the TRAPs group and the sIUGR II subgroup could be an indirect evidence for this presumption. The blood flow in the targeted vessels, completely from the donor twin or mainly from the retained larger co-twin, was not prone to be affected by the output from the reduced twin [8]. Blood flow disappearance indicated a complete blockage; hence, postprocedural exsanguination was avoided.

The incidence of brain damage and neurodevelopmental impairment in the reserved co-twin was reported to be 2/103 in a systematic review [30] and 5/74 in an observational cohort study after selective foeticide in MC pregnancies [32]. In this cohort, no neurodevelopmental retardation was found, except for mild ventriculomegaly in one pregnancy, and all children were observed to be doing well. The reason could be that delivery before 28 weeks was considered as an abortion in local hospitals and most of those newborns delivered before 28 weeks died without active treatment. Preterm birth as a consequence of PROM was considered as one of the main risk factors for adverse perinatal outcomes after foetal intervention [16, 31].

The limitation of this study lies in its retrospective nature. The initial case selection such as selective avoidance of sIUGR III and TTTS IV, leading to the small sample size in certain subgroups, may result in statistical bias. Moreover, some surgical details, such as placental penetration, entry of a twin sac and whether the reduced twin was the presenting twin, were missed. Another shortcoming is owing to the distance from the patients’ home to our hospital, most patients were followed up and delivered at local hospitals where viable preterm newborns had to be given up and most of the neurodevelopmental follow-up was merely assessed via telephone interview. Long-term follow-up with a standard cognitive and developmental scale is necessary.

## Conclusions

Compared to previous publications, this is the largest cohort study regarding the perinatal outcomes of RFA stratified by all possible indications. Furthermore, all of the procedures were conducted by one experienced operator and the same sonographer, thereby maximally decreasing the influence of the technique and measurement bias. In conclusion, the pregnancy outcome after RFA was found to be correlated to the indication, with the lowest survival rate in TTTS IV cases and the highest IUFD incidence in foetal anomaly cases affecting the anterior abdominal wall, followed by the sIUGR III cases. Selective RFA after 17 weeks may help prevent IUFD in DCTA pregnancies.

## Data Availability

All data included in this study are available upon request by contact with the corresponding author. No administrative permission was required to access the raw data.

## Abbreviations

RFA: Radio frequency ablation
IUFD: Intrauterine foetal demise
sIUGR: Selective intrauterine growth restriction
TTTS: Twin-to-twin transfusion syndrome
TRAPs: Twin reversed arterial perfusion sequence
IUFD: Intrauterine foetal demise
PROM: Preterm rupture of membrane
TOP: Monochorionic tramniotic
GA: Gestational age
MCDA: Monochorionic diamniotic
